# Molecular Targets and Strategies for Inhibition of the Bacterial Type III Secretion System (T3SS); Inhibitors Directly Binding to T3SS Components

**DOI:** 10.3390/biom11020316

**Published:** 2021-02-19

**Authors:** Julia A. Hotinger, Heather A. Pendergrass, Aaron E. May

**Affiliations:** Department of Medicinal Chemistry, School of Pharmacy, Virginia Commonwealth University, Richmond, VA 23219, USA; hotingerja@vcu.edu (J.A.H.); pendergrassha@vcu.edu (H.A.P.)

**Keywords:** type III secretion system, anti-virulence, inhibition, antibacterials

## Abstract

The type III secretion system (T3SS) is a virulence apparatus used by many Gram-negative pathogenic bacteria to cause infections. Pathogens utilizing a T3SS are responsible for millions of infections yearly. Since many T3SS knockout strains are incapable of causing systemic infection, the T3SS has emerged as an attractive anti-virulence target for therapeutic design. The T3SS is a multiprotein molecular syringe that enables pathogens to inject effector proteins into host cells. These effectors modify host cell mechanisms in a variety of ways beneficial to the pathogen. Due to the T3SS’s complex nature, there are numerous ways in which it can be targeted. This review will be focused on the direct targeting of components of the T3SS, including the needle, translocon, basal body, sorting platform, and effector proteins. Inhibitors will be considered a direct inhibitor if they have a binding partner that is a T3SS component, regardless of the inhibitory effect being structural or functional.

## 1. Introduction

The discovery and use of antibiotics have contributed to the overall increase in life expectancy throughout the world [1]. Notwithstanding the great success of antibiotic therapy, the over-prescription and misuse of broad-spectrum antibiotics have led to new challenges. Resistance to antibiotics threatens to make our current bacterial infection treatments ineffective [2]. One solution to the resistance problem is the use of anti-virulence therapeutics. This involves targeting the ability of pathogens to cause and sustain infections. Anti-virulence drugs would stop or slow infection while exhibiting minimal selective pressure [3]. One notable anti-virulence target is the type III secretion system (T3SS), a virulence factor used by pathogenic Gram-negative bacteria [4,5]. Some human pathogens that use T3SSs include, but are not limited to, *Bordetella* spp. [6], *Burkholderia* spp. [7], *Chlamydia* spp. [8], enteropathogenic and enterohemorrhagic *Escherichia coli* (EPEC and EHEC, respectively) [9,10,11], *Pseudomonas aeruginosa* [4], *Salmonella* spp. [5], *Shigella* spp. [12], *Vibrio cholerae* [13], and *Yersinia* spp. [14,15]. Pathogens targeting non-mammalian (*Aeromonas* spp. [16], *Edwardsiella* spp. [17], *Vibrio* spp. [18]) and plant hosts (*Erwinia* spp. [19], *Pseudomonas syringae* [20,21], *Ralstonia* spp. [20], *Xanthomonas* spp. [22]) are also found in nature. Given the conserved nature of the T3SS among pathogens, this target lends itself to the possible development of therapeutics capable of treating multiple infections, whether in human medical care, livestock upkeep, or agriculture.

**Figure 1:**
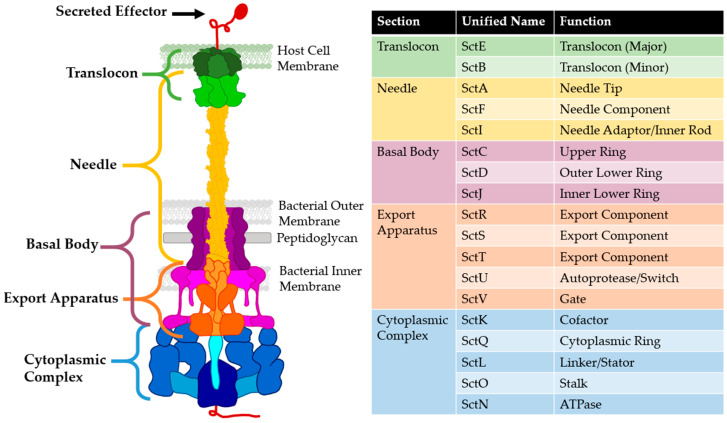
The general structure of the T3SS. **Left**: Cross-section of a prototypical T3SS with color-coded and labeled sections. **Right**: Table of unified names of T3SS structural components by section [23].

The T3SS is a protein complex that secretes proteins directly from the cytosol of the bacterial pathogen into the host cell via a tube spanning the bacterial and host membranes. Any protein that has been secreted by the T3SS is considered an effector protein. Some include the T3SS needle and translocon components in this distinction due to their secondary effects as virulence factors or an outdated convention [23,24,25,26], but in this review effector proteins will be considered any protein secreted by the completed T3SS. These molecular syringe-like injections have given the T3SS the nickname “the type III injectisome” [2,27,28,29,30,31]. The structure of the injectisome can be broken down into five major regions: the translocon, needle, basal body, export apparatus, and cytoplasmic complex (Figure 1). The translocon (Figure 1, dark green) is a protein hexamer pore that is formed in the host cell membrane that binds to the needle tip and allows secreted proteins to pass into the host [32,33,34,35]. The needle tip protein (Figure 1, light green) is also considered the translocon component by some but will be considered part of the needle in this review. The needle itself is composed of helical monomers (Figure 1, yellow) that form a tube-like structure after polymerization [31]. The needle differs in length from species to strain depending on the pathogen’s target host [36]. These variations will be discussed in the needle inhibition section.

The basal body is made of a dual ring system with the inner ring (Figure 1, light purple) spanning the inner bacterial membrane (IM) and the outer ring (Figure 1, dark purple) spanning the peptidoglycan layer (PG) and outer bacterial membrane (OM), anchoring the needle to the bacterial cell surface [37]. The export apparatus (Figure 1, orange) works with the cytoplasmic complex (Figure 1, blue) to sort, guide, and power secretion, and together are called the sorting platform [38]. The cytoplasmic complex contains an ATPase (Figure 1, dark blue) to power secretion. The sorting platform also functions as the recognition domain for effectors. It receives them from chaperone proteins and unfolds the effectors for secretion as folded proteins are too large to enter the needle (~2.5 nm inner diameter) [39,40,41]. Once the secreted proteins pass into the host cell, they elicit specific responses to reprogram the host’s machinery to enable colonization. In addition to reprogramming (*Salmonella* spp., *Shigella* spp.) [29,30], some effectors kill the target cells (*Yersinia* spp., *Pseudomonas* spp.) [31,39]. Individual pathogens can have upwards of 30 unique genes for effectors [42,43,44].

Many T3SS knockout strains have attenuated or eliminated virulence, making the T3SS an attractive anti-virulence target [32,36,40,45,46]. There have been no mammalian colonizing non-pathogenic bacteria found to use a T3SS, suggesting that agents targeting the T3SS will be selective for mammalian pathogens [47,48]. The highly conserved nature of the T3SS may also serve to provide a potential for cross-protection against multiple species of bacteria. There have been instances of inhibitors showing efficacy in vitro against multiple bacterial species and strains [25,49,50]. The extent of this cross-protection has yet to be investigated. This is potentially due to the high homology of the T3SS with the bacterial flagella. With this in mind, a general T3SS inhibitor may prevent the motility of commensal bacteria, resulting in a loss of many benefits to a pathogen-specific treatment. A common theme among T3SS inhibitor screening assays is a lack of toxicity to the pathogen [51,52]. This means the inhibitors do not exert selective pressure, potentially reducing the rate of resistance formation to these agents [53]. T3SS inhibitors are usually discovered through in vitro assays and are known as compounds that inhibit the secretion phenotype. Given the complex nature of the needle apparatus, the molecular targets of discovered inhibitors are often unclear. Although the importance of T3SS inhibitors for plant-targeting bacteria is of high importance and has many potential applications [54], the examples given in this review will focus on pathogens clinically relevant to veterinary and medical care. This review covers known and potential targets and strategies for direct inhibition of T3SS components.

## 2. Needle and Translocon

**Figure 2:**
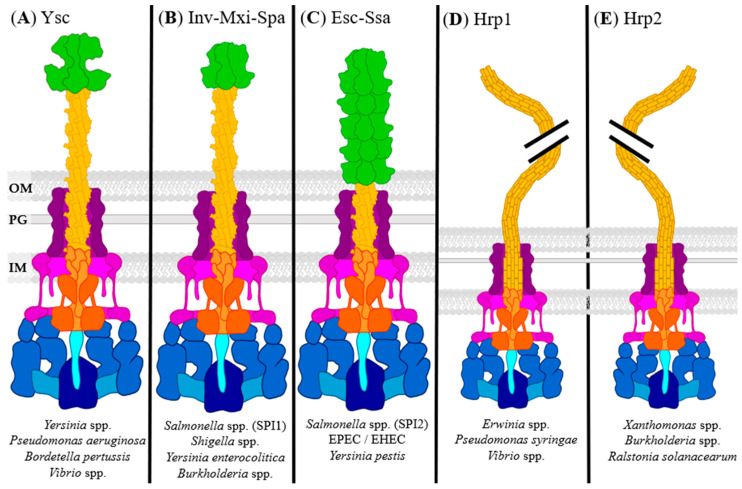
The 5 main groups of T3SS needles. (**A**) Ysc Group; (**B**) Inv-Mxi-Spa Group; (**C**) Esc-Ssa Group; (**D**) Hrp1 Group; (**E**) Hrp2 Group. Modified from [59]. Abbreviations: SPI1, *Salmonella* pathogenicity island; SPI2, *Salmonella* pathogenicity island 2; OM, bacterial outer membrane; PG, bacterial peptidoglycan layer; IM, bacterial inner membrane.

The type of needle presented in the T3SS complex depends on the target organism and the colonization style of the pathogen [55]. For example, T3SSs of plant pathogens, like *P. syringae*, have much longer needles (up to several µm) as they need to puncture through cell walls, while those infecting animals, like *Yersinia enterocolitica*, have shorter needles (~58 nm). Most T3SSs, however, have similar needle outer diameters, approx. 7 nm [56]. There are seven major groups of the T3SS, the five most common are shown in Figure 2 [55,56]. The four eukaryotic-targeting T3SS groups are Ysc, Inv-Mxi-Spa, Esc-Ssa, and Chlamydiales. They have similar needle lengths and base structure, but effectors and needle tip structure differ based upon their virulence needs. The plant pathogen groups, Hrp1, Hrp2, and RHIZ, are nearly identical in structure but vary in regulation and effectors more significantly [56]. Some pathogens encode multiple T3SS, often from different groups, with each having separate effectors. *S. enterica* uses two unique T3SSs encoded by *Salmonella* pathogenicity islands 1 and 2 (SPI1 and SPI 2, respectively). SPI1 encodes an Inv-Mxi-Spa group T3SS for invasion, and SPI2 encodes an Esc-Ssa group T3SS for intracellular survival [57]. The differences in T3SS group structure are essential to understand when inhibiting specific pathogens, especially when targeting the needle and tip as they are the most variable between the groups.

Ysc group T3SSs are seen in the largest variety of eukaryotic pathogens, including *Yersinia* spp. [14,15], *P. aeruginosa* [4], *Bordetella pertussis* [6], *Photorhabdus luminescens* [58], *Aeromonas* spp. [16], and *Vibrio* spp. [13,18] (Figure 2A). The group is named after the Ysc pathogenicity island of *Y. pestis*, the causative agent of bubonic plague. It has a distinctive needle tip in each of its pathogens, creating a highly selective target for anti-virulence therapies. The Ysc group T3SS secretes effectors that assist in adhesion, resist phagocytosis, and prevent inflammatory responses.

The Inv-Mxi-Spa group is named after the Inv-Spa T3SS of *Salmonella enterica* and the Inv-Mxi T3SS of *Shigella* spp. [5,12] (Figure 2B). This needle type is involved in secreting effectors that trigger bacterial uptake and cellular invasion and correlates with these pathogens being intracellular. The SPI1 T3SS of *S. enterica* was the first to be observed and is often considered the prototypical T3SS [5]. Other notable pathogens utilizing this T3SS are *Y. enterocolitica* biogroup 1B [60], *Sodalis glossinidus* [61], *Burkholderia* spp. [62], and *Chromobacterium violaceum* [63].

Esc-Ssa characteristically has a needle tip longer than the needle itself, often called the filament due to its length (Figure 2C). The archetypal T3SS for this group is the Esc T3SS expressed in EPEC and EHEC, used primarily for adhesion and persistence [9,10,11]. In these pathogens, the filament is hollow, with an interior diameter of ~25Å, which is only large enough to allow passage of linear or partially unfolded proteins [64]. It has been proposed the filament evolved to help EPEC/EHEC reach their target intestinal epithelial through a mucus layer that lines the intestine The SPI2 encoded T3SS of *S. enterica* is Esc-Ssa group and is used after the invasion has occurred and used to prevent endocytic trafficking and phagosome maturation, thus allowing *S. enterica* to survive within macrophages [57]. Other pathogens encoding an Esc-Ssa group T3SS include *Edwardsiella* spp. [17], *Citrobacter rodentium* [65], and *Yersinia* spp. (nonfunctional) [66].

The Chlamydiales group T3SS (not pictured) is the last eukaryotic pathogen T3SS and is not well studied in comparison to the Ysc, Inv-Mxi-Spa, and Esc-Ssa groups. It is only present in *Chlamydia* spp. and has highly specific expression and secretion conditions, making it difficult to study in vitro [8]. The Chlamydiales T3SS is also not encoded on a single pathogenicity island like the other T3SS, making it difficult to study genetically as well. It differs from the other eukaryotic-targeting groups structurally by extra length in the N-terminus of both basal body rings and possibly lacking a stalk protein. This group secretes effectors that assist in intracellular lifestyles [8].

Hrp1 and Hrp2 groups are the prototypical plant pathogen T3SSs, each having a long, flexible pilus instead of a rigid needle (Figure 2D,E). They are highly similar in structure and as such are separated based mainly upon loci organization and regulatory systems, but the distinction is also visible phylogenetically [67]. Regardless of the structural similarities, each pathogen varies widely in effector number and composition and is highly specialized to its specific host. The Hrp1 T3SS found in *Erwinia* spp. [19] and *P. syringae* [20,21] is activated by HrpL, a sigma factor of the extracytoplasmic function subfamily. On the other hand, the Hrp2 T3SS of *Ralstonia* spp. [20] and *Xanthomonas* spp. [22] is activated by HrpB, a member of the AraC family [68]. Hrp1 and Hrp2 T3SSs also appear as secondary or tertiary T3SS in eukaryotic-targeting bacteria such as *Vibrio parahaemolyticus* and *Burkholderia* spp. [69,70]. Although the function of this secondary T3SS is unknown, it has been proposed that it has a role in cell-cell interaction within a bacterial population [69].

The Rhizobiales (RHIZ) group T3SS (not pictured) is highly similar to the other plant-pathogen T3SSs in structure, but not in function. The RHIZ T3SS is found only in *Rhizobium* spp., *Mesorhizobium loti*, and *Bradyrhizobium japonicum* [71]. Rhizobiales form a symbiotic relationship with their plant host through the formation of root nodules. These RHIZ T3SS can assist in nodule formation or favor the host’s defensive reaction and prevent colonization [72].

### 2.1. Needle Formation Inhibition

**Figure 3:**
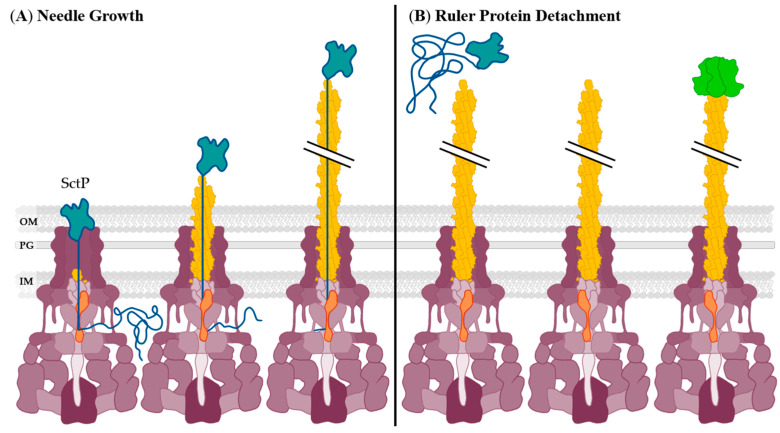
Ruler protein measuring the length of T3SS needle polymerization. (**A**) SS interacts with SctC to signal needle component secretion (SctF, yellow). Ruler protein (SctP, blue) assisting in SctF polymerization. SS acts as a cap to the needle and LS interacts with SctU (orange) as it is threaded through the export apparatus. SctP reaches the full length and SctF polymerization halts; (**B**) LS is released from SctU and SctP is secreted. SctU has a conformational change that activates the injectisome to switch to middle secretion, including the needle tip (green). Modified from [74]. Abbreviations: OM, outer membrane; PG, peptidoglycan; IM, inner membrane.

The length of a T3SS needle is essential to its function and as such is regulated by a specific mechanism; one accepted mechanism is an “infrequent ruler model,” proposed by Moriya et al. [73]. This involves the “ruler protein”, SctP, measuring the length of the needle as it polymerizes (Figure 3). SctP has an N-terminal unfolded “length sensing” (LS) domain that is recognized by SctU, the autoprotease, that determines the length of the needle. This length is specific to each pathogen’s need. For example, in EHEC EscP is ~140 amino acids while in *Yersinia* spp. YscP is >500 amino acids [74]. The globular C-terminus is called the substrate-switching (SS) domain and interacts with extracellularly exposed SctC, the basal body upper ring, initiating needle formation [74].

To start the needle formation process, oligomers of protein SctI form an inner rod structure sometimes called the needle adaptor, which is essential to type III secretion [75]. It interacts with the SctC and SctU [76]. The needle itself is composed of oligomerized SctF and is required for the successful secretion of effectors and the rest of the structural components of the T3SS [77,78]. As SctF is secreted and begins to polymerize it pushes up the SS domain of SctP, which then acts as a cap to the growing needle (Figure 3A). Through direct interaction with SctF monomers, SctP moves through the interior of the needle complex, and secretion of SctF stops after the needle reaches the same length as SctP. At this point, there is an interaction between SctU and SctP result in the secretion of SctP (Figure 3B). This interaction is proposed to cause the conformational change in the SctU proteins responsible for changing the specificity of effector binding to allow middle and late-stage secretion [73,74].

After the needle is assembled, the secretion of the final structural components begins. SctA is the needle tip of the T3SS and varies widely among different pathogens. For example, most needle tips are pentamers of SctA [79], while EPEC SctA monomers interact through a coiled-coil domain to form a homo-oligomer filamentous structure, which builds from interactions with the needle, EscF [77,80,81]. Some consider the needle tip to be the hydrophilic component of the translocon due to this important adhesion to the hydrophobic translocon components, SctE and SctB. Deletion of SctA inhibits bacterial attachment, making it integral to the T3SS [82]. SctE and SctB are then secreted and form a hetero-oligomeric donut-shaped structure within the eukaryotic host cell membrane, called the translocon [83]. SctA serves as a scaffold for pore formation by SctE and SctB [84]. SctE acts as the major subunit, able to form pores within the host membrane itself, while SctB is the minor subunit, unable to form pores alone [82]. The pore formation of SctE and SctB to create the translocon is the final step in the construction of the T3SS.

A needle length that is too long could inhibit intimate attachment and/or internalization processes. A stunted needle length can prevent attachment due to mucosal lining or host extracellular proteins physically blocking the bacteria from coming close enough. In the case of plant pathogens, a stunted needle can prevent the needle from reaching the cytoplasm of the host. The simplest method of needle inhibition is the prevention of needle subunit polymerization. This can be done by physically preventing the needle subunits from binding to each other, inducing incorrect folding of the subunits, or modulation of conditions required for correct needle polymerization.

It is common for there to be a coiled-coil domain in the needle tip and translocon, a structure composed of an amphipathic secondary α-helix. The presence of this domain is hypothesized to play a role in multimeric oligomerization, molecule recognition, and potential interaction between bacterial and eukaryotic proteins upon translocation. In pathogenic *E. coli*, the proteins responsible for forming the filament (EspA) and needle (EscF) have coiled-coil domains, as does the chaperone for EspA (CesA). To analyze the potential inhibitory effects of these domains on T3SS function, coiled-coil mimetics were synthesized based on the amino acid (AA) sequences of EspA, EscF, and CesA (Table 1). The ability of these peptides to inhibit T3SS activity was quantified based on hemolysis of red blood cells (RBCs) in the presence of EPEC [85]. T3SS-mediated hemolysis was inhibited by 95% in the presence of Coil A or Coil B at 0.16 mM. Coil AB1 and Coil AB2 showed less potent inhibition of between 10% and 20% at 0.14 mM. Coil C and Coil D did not exhibit inhibitory effects to a major extent. CesA1 did not inhibit hemolysis, but CesA2 inhibited hemolysis by ~50% at 0.23 mM. With the growing prevalence of peptide drugs approved by the FDA [86], and given that these coiled peptides target extracellular proteins, they show promise for use as pharmaceutical agents.

The salicylidene acylhydrazides (SAs) are a well-characterized structural class of small molecule T3SS inhibitors. They were originally discovered as inhibitors of the *Y. pestis* T3SS [87] but have since been implicated as T3SS inhibitors for *Chlamydia* spp. [88,89], *Salmonella enterica* serovar Typhimurium (*S*. Typhimurium) [90]. *Shigella* spp. [91], EHEC [92], *Xanthomonas oryzae* [93,94], and *Erwinia amylovora* [95]. Veenendaal et al. investigated whether a group of SAs prevented expression of or destabilized the inner lower ring and/or the needle subunit of the T3SS in whole-cell extracts of bacteria exposed to the compounds. Western blotting showed that the levels of these proteins remained in all samples and controls [91]. However, they noted a possibility that the compounds affected the assembly of the T3SS without affecting the levels of its protein components. Electron microscopy was used to visualize the macromolecular T3SS in *Shigella* cells from cultures grown in the presence of the inhibitors after cytoplasm removal. There was a 30-40% decrease in injectisomes per cell in all compound-treated samples, as well as an increased number of injectisomes without needles or with truncated needles, giving strength to the needle subunit-related inhibition mechanism theory [91].

**Figure 4:**
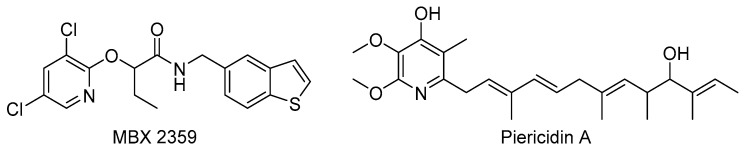
Structures of MBX 2359 and piericidin A.

In a screen of over 80,000 compounds done by Aiello et al., the phenoxyacetamide scaffold was discovered as a potent T3SS inhibitor class [49]. The assay allowed for visualization of inhibitory effects on ExoT expression, a *P. aeruginosa* effector, using a fused *luxCDABE* operon to the *exoT* gene. The inhibition of secretion of an ExoS-β-lactamase fusion, an effector in *P. aeruginosa*, as well as native ExoS was observed via sodium dodecyl sulfate-polyacrylamide gel electrophoresis (SDS-PAGE) [49]. The same research group later did mutational studies using five phenoxyacetamide analogs, including MBX 2359 (Figure 4), to determine the target of these inhibitors. They selected gentamicin-resistant clones of ethyl methanesulfonate-mutagenized MDM1710 in the presence of MBX 2359. Mutations in the *pscF* gene, the needle subunit in *P. aeruginosa*, were sufficient to rescue T3SS activity in the presence of MBX 2359, while still susceptible to other classes of inhibitors [96]. MBX 2359 has since been used in vivo to show that T3SS inhibition protects against *P. aeruginosa* abscess formation in mice [34]. This inhibitor class is also active against *Yersinia* and *Chlamydia* T3SS [49].

In 2014, Duncan et al. performed a high throughput screen to discover new inhibitors of the *Y. pseudotuberculosis* T3SS and uncovered piericidin A’s inhibitory activity (Figure 4) [97]. SDS-PAGE analysis indicated that secretion of YopE was decreased by 65% at 71 μM piericidin A. Piericidin A was also shown to potently inhibit translocation of YopM into Chinese hamster ovary (CHO) cells at 75% at 71 μM. Although piericidin has a known antibacterial target (Complex I), an alternative Complex I inhibitor, rotenone, has no TTSS inhibitory activity, indicating the TTSS inhibitory activity of piericidin A may be independent of complex I inhibition [98]. The mechanism of action of piericidin A as a TTSS inhibitor is not known, but evidence suggesting inhibition of needle formation has recently been discovered [99]. Inhibition of the T3SS by piericidin A decreased formation of Ysc-type needle units on the surface of *Y. pseudotuberculosis* without interfering with gene expression, indicating the mechanism is directly related to needle assembly [99]. Further work to identify the exact T3SS inhibition target of piericidin A would aid in the rational design of more potent analogs that selectively inhibit the T3SS without antibiotic effects related to Complex I binding.

### 2.2. Needle Tip and Translocon Inhibition

**Figure 5:**
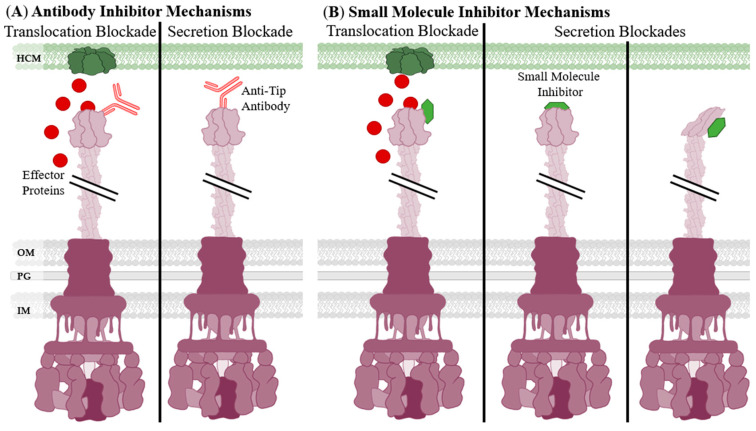
Inhibitors interacting with the T3SS needle tip. (**A**) Antibody inhibitors; **Left**: Antibody binding to the needle tip preventing binding to the translocon; **Right**: Antibody binding and physically preventing the secretion of effectors; (**B**) Small molecule inhibitors; **Left**: Translocation blockade caused by the inhibitor binding to the needle tip; **Middle**: Small molecule binding and physically preventing effector secretion; **Right**: Conformational change of needle tip protein caused by small-molecule binding. Image modified from [102]. Abbreviations: OM, outer membrane; PG, peptidoglycan; IM, inner membrane; HCM, host cell membrane.

Inhibitors, whether small molecule or antibody, targeting the T3SS needle tip will likely adopt the secretion blockade mechanism of pathogenesis prevention [100]. Secretion blockades have two variations: true secretion blockades and translocation blockades (Figure 5). Concerning the T3SS, translocation is defined as secretion directly into a host cell while secretion is the expulsion of protein through the T3SS needle [101]. True secretion blockades occur when the inhibitor physically prevents the secreted proteins from exiting the needle either with itself blocking the pore or changing the conformation of the tip protein (Figure 5B). When designing anti-needle tip inhibitors, a true blockade style inhibition is desirable because the secreted proteins are not released.

When an inhibitor binds to the needle tip without blocking the pore it can still prevent binding of the tip to the translocon, creating a translocation blockade [102]. Many effector proteins cannot pass through the membrane after secretion, allowing them to stay within the host [26]. When the needle tip is prevented from binding from the translocon by an antibody or small molecule, then effectors are secreted into the intercellular space where they can no longer enter the host cell (Figure 5). The translocation blockade is considered a cause of unproductive use of effectors or “wasting effectors” that results in less virulence.

In 1958 researchers noticed that V antigen was present in pathogenic strains of *Yersinia*, but not in non-pathogenic strains [103]. V antigen was later determined to be LcrV, the needle tip protein of the *Y. pestis* T3SS [104,105]. Antibodies targeting LcrV were sufficient to prevent translocation of effector proteins by the *Y. pestis* T3SS [106]. Ivanov et al. confirmed that anti-LcrV mAbs were sufficient to directly prevent the secretion of Yop effector proteins [107]. Their work provided a foundation to show that anti-needle tip Abs were acting as a secretion blockade and not a translocation blockade (Figure 5) [100]. One example of this phenomenon is a specific anti-LcrV Ab blocking the apoptotic action of LcrV, the *Yersinia* spp. needle tip, against human T-cells [108].

Some inhibitors of the needle tip have gone into human trials. One of these therapies, a bi-functional anti-PcrV and anti-Psl mAb for the treatment of *P. aeruginosa* infections called MEDI3902, showed a dose-dependent survival increase and a decrease in bacterial load in both rabbit and mouse *P. aeruginosa* challenge models. MEDI3902 also reduced lung inflammation caused by bacterial colonization [109,110,111,112]. MEDI3902 performed well in phase I clinical trials for the treatment of chronic airway *P. aeruginosa* infections but did not show high enough effectivity in phase II trials in the US [113,114]. Despite this setback, work on MEDI3902 has continued. Le et al. showed MEDI3902 was effective as a treatment and prophylactic for acute blood and acute lung *P. aeruginosa* infections. Combination therapy with a subtherapeutic dose of the antibiotic meropenem enhanced effectivity of MEDI3902 [115].

**Figure 6:**
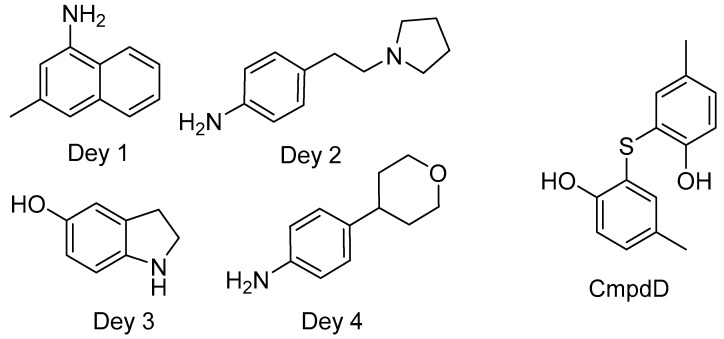
Structures of Dey 1-4 and CmpdD.

As the needle tip protein is essential to the T3SS, Dey et al. screened nearly 300 compounds via surface plasmon resonance to identify small-molecules that bind to IpaD, the needle tip in *Shigella* spp. [116]. Four small molecules were identified that have scaffolds based on quinoline, pyrrolidine aniline, hydroxyindole, and morpholinoaniline (Figure 6, termed Dey 1-4, respectively, in this review for brevity and clarity). They further validated their findings by determining the residues and surfaces of IpaD interacting with the compounds by saturation transfer difference (STD) and titration NMR methods. Three potential binding pockets were discovered, all of which are involved in protein-protein interactions of IpaD with other T3SS components [116]. Before their screen, the only small molecules known to bind to IpaD were the bile salt sterols deoxycholate, cholate, chenodeoxycholate, and taurodeoxycholate [117,118,119]. The bile salt compounds have been shown to bind in one of the binding pockets identified in the Dey study [116]. When bound, they induce a conformational change in IpaD that allows interactions with IpaB during translocon assembly [120].

Jessen et al. have shown that the T3SS inhibitor compound D (termed cmpdD in this review for brevity and clarity, Figure 6) inhibits T3SS secretion in *Y. pestis*, *Y. pseudotuberculosis*, and *P. aeruginosa*, via interaction with a translocon component [25]. They found YopD, the minor translocon component in *Yersinia* spp., was specifically implicated in cmpdD’s inhibition mechanism. The effector and structural proteins YopB, YopD, YopK, YopM, YopE, and LcrV secretion were inhibited, except for LcrV, indicating that an intact needle was present before inhibition took place. They then tested YopB and YopD null strains of *Y. pestis* for cmpdD-mediated inhibition of Yop secretion and found that YopD plays a role in cmpdD’s inhibition mechanism. Secretion assays in the presence and absence of calcium were performed. Interestingly, cmpdD was only effective as an inhibitor in a calcium-depleted environment. This enhances the theory that cmpdD-mediated inhibition occurs after the needle is complete [25], suggesting the formation of a secretion blockade by cmpdD.

**Figure 7:**
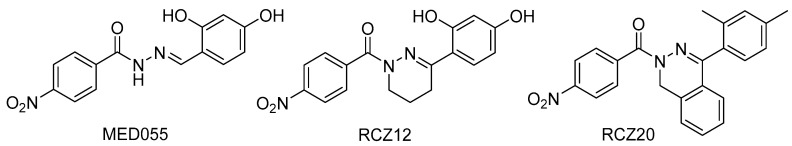
Structures of salicylidene acylhydrazides MED055, RC12, and RC20.

The SAs are a broad category of T3SS inhibitors that are promiscuous binders [121]. Zambelloni et al. designed a new class of SAs using MED055 (Figure 7) as a scaffold and incorporating the imine moiety into two different hydrazine-containing heterocycles, 1,4,5,6-tetrahydropyridazine (THP) and 1,2-dihydrophthalazine (DHP) [122]. Of the eight new compounds, the sulfonyl di-hydroxyl derivatives (RCZ12 and RCZ20, Figure 7) had the strongest EHEC T3SS secretion inhibition profile with multiple effectors undetectable when in the presence of 200 µM of either compound. They performed pull-down assays to determine the molecular target of RCZ12 and RCZ20. EspD was the only T3SS-related protein to show affinity to the compounds, although some off-target cellular targets, notably 2-oxoglutarate dehydrogenase and elongation factor Tu, were identified. The selectivity of the two compounds was, however, significantly better than MED055 [121]. The affinity data was further supported by mechanistic studies that determined the coiled-coil domain 1 of EspD as a binding site of both RCZ12 and RCZ20 [122].

### 2.3. Structural Chaperone Inhibition

Chaperone proteins are classified as proteins that help with conformational changes and/or folding of proteins after transcription. A pilot protein assists in the localization of proteins. In the case of T3SS chaperones, they perform both duties, prevention of folding the protein and then localizing it to the T3SS to be secreted [123,124]. Most chaperones function as dimers [124,125]. Associations between a chaperone and its substrate are dependent on a specific AA sequence present on the N-terminus of the substrate called the chaperone binding domain (CBD) [123,126]. The CBD is recognized by the sorting platform and then the substrate is released, allowing secretion. The chaperone is then free to receive another protein and continue the cycle [127].

There are three classes of chaperones, class I, class II, and class III. The first class is dedicated to chaperones that transport effector proteins and are further separated into IA and IB. These chaperones will be discussed in more detail in the effector inhibition section of this review. The second and third classes of chaperones are involved in transporting structural protein components of the T3SS including the needle subunit, needle tip, and translocon components (Table 2). Class II chaperones contain tetratricopeptide (TPR) repeats that are required to bind and secrete the translocon components. Homodimerization allows for these TPR to form a “cupped palm” groove that the translocon components then bind to [128].

Class III chaperones are heterodimeric, working in pairs, and bind to the needle protein subunits, typically in a 1:1:1 ratio. This is done to prevent polymerization of the needle within the bacteria before secretion [128]. For example, EscF, the needle subunit in EPEC, is transported to the T3SS by co-chaperones EscE and EscG, which form interactions with the bacterial membrane while in complex with EscF. Null mutants of EscE and EscG do not secrete multiple T3SS proteins, further implementing EscF and its chaperones as a necessity for type III secretion [129]. Of note, some bacterial species have subunits of a differing conformation such as EspA, the needle tip in EPEC, that require only one chaperone, CesA in this case, to prevent polymerization within the cytosol [130,131,132]. Needle tip proteins of the Inv-Mxi-Spa family contain a chaperone-like domain and are proposed to be self-chaperoning [133].

Inhibiting the chaperones of structural components of the T3SS will have similar effects to inhibition of the structural component itself. Class II chaperone inhibitors will prevent the translocon from entering the injectisome. This will cause an unproductive use of effectors as there will be no translocon pore for the effectors to translocate through. Phenotypically distinguishing between a component inhibitor or an inhibitor of its chaperone is very difficult, meaning many class II and III chaperone inhibitors will go unidentified [153]. In one study, a small molecule T3SS inhibitor, cmpdD (Figure 6), was thought to be interacting with YopD, a translocon component, directly to prevent secretion [25]. This hypothesis was brought into question, however, when the expression of YopD in the ΔyopD *Y. pestis* strain did not restore secretion inhibition in the presence of cmpdD. Only co-expression of yopD and LcrH, the class IA chaperone of YopD, was able to restore cmpdD’s ability to inhibit secretion. Together these results suggest the YopD/LcrH complex may be involved in the mechanism of cmpdD [25].

**Figure 8:**
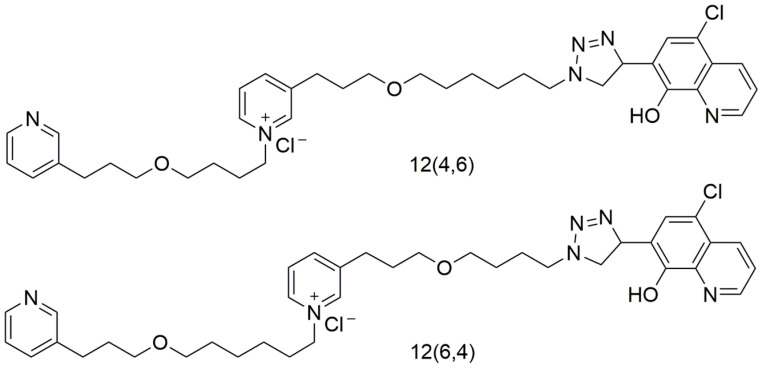
Structure of 12(4,6) and 12(6,4).

Recently, the co-chaperone nature of class III chaperones has been exploited by Ngo et al. to design protein-protein interface inhibitors. They chemically linked two smaller bioactive molecules to design inhibitors that will disrupt the interaction between the co-chaperones, PscG and PscE, that stabilize PscF, the *P. aeruginosa* needle subunit protein [154]. They found two compounds, 12(4,6) and 12(6,4) (Figure 8) that were able to disrupt the interaction between PscG and PscE as well as prevent the secretion of effectors ExoT, ExoS, and translocon component PopD in a dose-dependent manner. The compounds also did not affect growth, flagellar movement, or biofilm formation, indicating specificity to the T3SS. 12(4,6) and 12(6,4) also provided partial protection from *P. aeruginosa* infection in a *Galleria mellonella* model [154].

## 3. T3SS Base: Basal Body, Export Apparatus, and Cytoplasmic Complex

**Figure 9:**
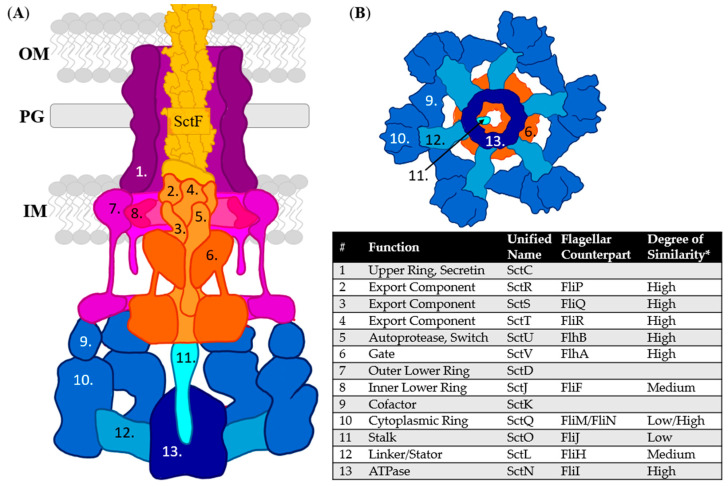
Structure of the T3SS basal body, export apparatus, and cytoplasmic complex with a list of unified protein names and functions. (**A**) Vertical section of the T3SS basal body and sorting platform. (**B**) A bottom-up view of the T3SS sorting platform. Abbreviations: OM, outer membrane; PG, peptidoglycan; IM, inner membrane. *Degree of similarity between flagellar and injectisome components in *Y. enterocolitica*: high, *E* < 10^−5^; medium, 10^−5^ < *E* < 0.01; low, *E* > 0.01 [155].

The base of the needle assembly anchors the T3SS into the inner and outer membranes (IM and OM, respectively) of the bacterial cell. The basal body of the T3SS contains two major components, the lower ring attached to the IM and the upper ring spanning the periplasm and OM (Figure 9, Purple). The export apparatus is also occasionally included in the basal body as it is located within the lower ring (Figure 9, Orange). In this review, however, it will be considered a separate component due to its function as a filter; determining the order of effectors to be secreted. The cytoplasmic complex (Figure 9, blue) contains an ATPase (Figure 9, dark blue) that powers secretion. The cytoplasmic complex and export apparatus together are often called the sorting platform as they determine order and rate of secretion as well as power it with the ATPase.

One notable downside to targeting the base of the T3SS is that it is highly conserved among other secretion systems and flagella [155,156]. One particularly poignant example of this is the export apparatus components, all of which have an *E* < 10^−5^ with their flagellar counterparts (Figure 9) [155]. The expectation or E-value is *E*(*b*) ≤ *p*(*b*)*D*, where *D* is the number of sequences in the database and *p(b)* is the probability of two sequences being aligned [157]. Other than direct correlation to flagellar components, there are also issues with relation to other secretion systems. SctC belongs to a family of pore-forming proteins called secretin. Secretins are also found in type IV pili (T4P), type II secretion systems (T2SS), Flp pili encoded by the tight adherence (Tad) system, and the extrusion machinery of filamentous phages [158,159]. In each group of T3SS, the SctC has developed from the secretin of different secretion systems. For example, the majority of T3SS groups obtained their SctC from the T4P, while Chlamydial T3SS obtained their SctC from the T2SS [156]. These similarities can lead to off-target effects on commensal bacteria. Multiple T3SS inhibitors are known to affect flagella [160,161,162].

### 3.1. Formation Inhibition

**Figure 10:**
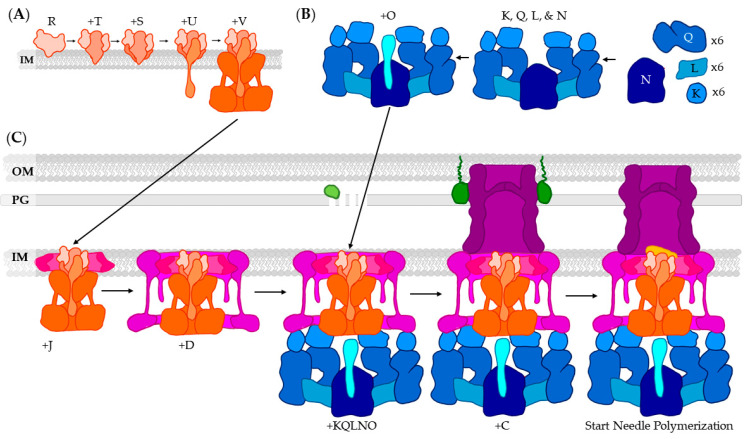
Assembly of the injectisome base. Protein names are abbreviated by their conserved name while omitting “Sct” from the beginning. (**A**) Independent formation of the export apparatus within the bacterial IM; (**B**) Formation of the cytoplasmic complex; (**C**) Basal body formation around the export apparatus and connection of cytoplasmic complex. Image modified from [163]. Abbreviations: OM, outer membrane; PG, peptidoglycan; IM, inner membrane.

The formation of the T3SS base occurs in three steps, the first two, export apparatus and cytoplasmic complex formation, happen simultaneously while the third, basal body formation, is sequential (Figure 10). The inhibition of any one of the steps in the T3SS base formation could prevent the completion of a functional needle and therefore the secretion of effectors into the host cell. This implies the design of protein-protein interaction inhibitors may be the best fit for this purpose. Genetic modulators that reduce the number of a particular component, thereby reducing the number of functional T3SS, are also a valid strategy, but outside the scope of this review. The components of the T3SS base are also the most conserved among T3SS proteins, making them an attractive target if a multi-species T3SS inhibitor is desired.

The export apparatus is formed in a highly ordered manner, from the most internal components to the more exterior: SctR, SctT, SctS, SctU, then SctV (Figure 10A) [163]. SctR is the central component of the export apparatus and initiates the assembly of the rest of the T3SS through interactions with other structural components [164]. The role of SctT is not well understood, but it has been identified as a T3SS protein based on sequence homology among multiple bacterial species [165]. When either of the two putative transmembrane domains of SctS are replaced by hydrophobic residues, the T3SS becomes non-functional, indicating SctS may play a role in adjustments of protein orientation within the base [166]. SctU is an autoprotease often called the switch of the export apparatus. The self-cleavage of SctU is necessary for infection of host cells [167]. In addition, an absence of SctU cleavage resulted in a decreased accumulation of chaperones on the IM. This suggests that the autoprotease activity of SctU contributes to the successful docking of effector/chaperone complexes [167]. SctV, the gate protein, is the largest of the export apparatus proteins and is homologous with flagellar protein FlhA, which facilitates the transport of sodium ions and protons [168]. SctV is proposed to function as a proton/protein antiporter [169]. Once the export apparatus is complete, the basal body will begin to form around the apparatus (Figure 10C) [163].

The cytoplasmic complex is not formed in any particular order and the components, SctK, SctQ, SctL and SctN, can associate at will (Figure 10B). This has the exception of the stalk protein, SctO, that associates last and can even associate after the cytoplasmic domain has bound to the basal body [163]. SctO stabilizes the oligomerization of the ATPase and helps prevent ATP-mediated dissociation. SctL mediates interactions between the ATPase, SctN, and the cytoplasmic ring, SctQ [170,171]. In plant pathogens, such as *P. syringae*, there are two proteins, termed HrcQ_A_ and HrcQ_B_, that interact to form the cytoplasmic ring [172]. Pull-down studies indicate that SctL, the linker, binds to the needle tip, SctA, and stabilizes it in the bacterial cytosol as a secondary function [132]. The final component, SctK, binds within the inner diameter of the cytoplasmic ring [171]. While the function of SctK is thought to be primarily structural, functional analysis of an SctK knockout indicates it is critical for effector secretion [171].

After export apparatus completion, first the inner lower ring, SctJ, and then the outer lower ring, SctD, will oligomerize to form a ring around the export apparatus and span the bacterial membrane (Figure 10C) [163,173]. Simultaneously, a lytic glycosylase (Figure 10, light green) is employed to cleave bonds within the PG layer, allowing base subunits to assemble. Overexpression of lytic glycosylase leads to cell lysis, and mutation in its catalytic residues results in a non-functional T3SS [174]. The lower ring components are necessary for the assembly of the upper ring, as null mutations result in decreased oligomerization SctC, the monomer of the upper ring, and a member of the secretin family of proteins [159]. After the lower ring is complete, the completed cytoplasmic complex can bind at any time. Pilotin (Figure 10, dark green) then assists in upper ring binding and pore formation before leaving [159]. Once all of these have been correctly assembled, the needle formation process can begin [163,175,176].

**Figure 11:**
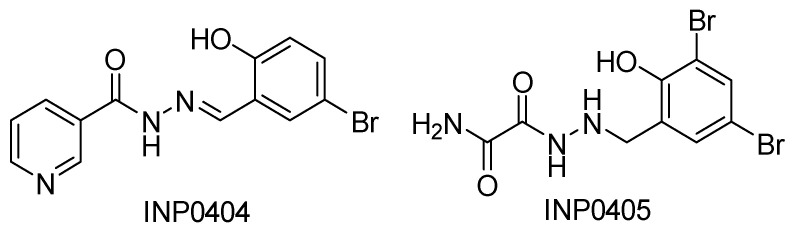
Structure of SA inhibitors INP0404 and INP0405.

In the absence of the export apparatus, there is a marked decrease in the base assembly, leading to an overall reduction in functional T3SSs [163]. In a study by Martinez-Argudo et al., *S*. Typhimurium mutants were isolated that were resistant to the SA inhibitors, INP0404, and INP0405 (Figure 11), allowing the determination of their target [162]. The mutants contained mutations affecting the gate protein (SctU), *fadB* (the γ-subunit of fatty acid oxidation complex), and the ATPase. One mutation to the ATPase caused the deletion of the first five amino acids, resulting in an inability to bind effectively to other components of the cytoplasmic complex. This mutation was also found to affect flagellar formation and motility. The point mutation affecting SctU was insufficient to cause disruptions in flagellar function, implying it is more specific to the T3SS inhibitory effect of INP0404 and INP0405. The overall expression levels of SctU were unchanged in the presence of the mutation, but the mutation did lead to a decrease in protein in the periplasm. Together these imply these inhibitors’ mechanism is directly interacting with the base of the T3SS, possibly binding to multiple components [162].

### 3.2. Basal Body: The Periplasmic Gate

**Figure 12:**
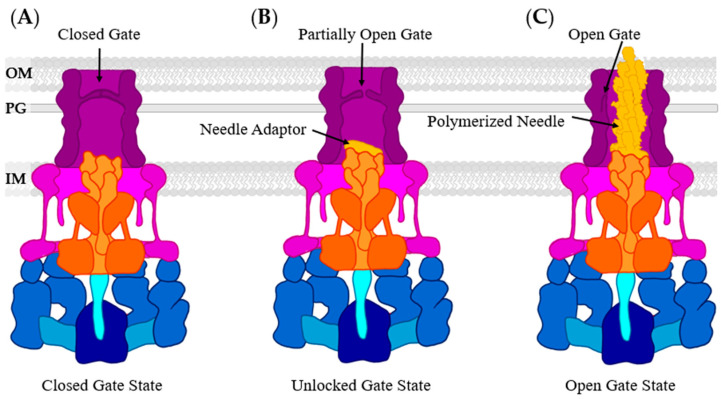
Periplasmic gate conformational states. (**A**) Closed gate state; (**B**) Unlocked gate state; (**C**) Open gate state. Image modified from [177]. Abbreviations: OM, outer membrane; PG, peptidoglycan; IM, inner membrane.

When mentioning the “gate” of the T3SS, there are two possible meanings. The major export apparatus protein, SctV, is often called the gate due to its function while the T3SS is operational. The other “gate” in question is called the periplasmic gate and is located within the upper ring, SctC, of the basal body and is involved in needle formation. The periplasmic gate has three conformations: closed, unlocked, and open (Figure 12) [177]. SctC is natively in the closed conformation as it associates with the lower ring of the basal body. The gate is locked via N3 domain β-hairpin stabilizing interactions. Needle adaptor proteins enter the canal of SctC and remain collapsed without lateral packing. After the subunits enter the canal, the β-hairpin interactions are destabilized, “unlocking” the gate and allowing it to partially open. Polymerization of needle subunits begins, and subsequent polymerization pushes the arms of the gate open and into the sides of the canal [177].

An effective mechanism to preventing secretion could be a conformational change in SctC that prevents periplasmic gate opening, ultimately resulting in no needle formation. As most inhibitors are discovered in phenotypic screens, there are no current inhibitors known to produce this effect via this mechanism. These inhibitors, however, could easily be mistaken as those interacting with needle subunit proteins as the phenotype would be similar. Notwithstanding this setback, there have been multiple inhibitors shown to interact with SctC and inhibit the functionality of the T3SS. One inhibitor class that may be interacting with periplasmic gate function is the salicylidene acylhydrazides.

**Figure 13:**
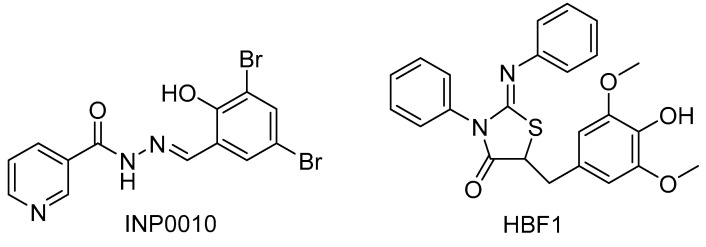
Structure of SA INP0010 and thiazolidinone HBF1.

Inhibition of the T3SS with SAs results in a decrease in injectisomes per cell as well as an increased number of injectisomes without needles or with truncated needles [91]. Recent studies have shown that a group of nine SAs inhibit both the T3SS-1 and T3SS-2 of *S*. Typhimurium, as well as decreasing flagellar motility [161]. The most potent SA that showed dose-dependent inhibition was INP0010 (Figure 13). This suggests that SAs if acting on a T3SS component directly, will be binding to a conserved factor among all three of these structures. Although export apparatus components may seem the likely target due to their high homology with flagellar components [155], the upper ring of the basal body has been identified as the potential target. This is because the N-terminus of SctC/secretin in the T3SS-1, T3SS-2, and flagella in *S*. Typhimurium share even higher homology [161].

The thiazolidinones have also been shown to interact with SctC. 2-Imino-5-arylidene thiazolidinone (Figure 13) was first discovered in a virtual screen to discover inhibitors of *S*. Typhimurium T3SS [178]. From this point onward termed HBF1, this inhibitor prevented the secretion of effectors SipA and SspH1 while not inhibiting bacterial growth or flagellar motility. HBF1 was proposed to bind to PrgH, the outer lower ring of the basal body in a way that prevented its alkaline phosphatase activity as well as binging to the upper ring. This was due to a lack of T3SS protein expression changes, examined via western blot, but a significant decrease in functional basal body and needle formation was observed [178]. Felise et al. found HBF1 was able to inhibit secretion of Yop effectors in *Y. enterocolitica*, indicating again that a highly conserved structural T3SS protein was involved in its mechanism of action. They also proved HBF1′s ability to prevent T3SS-dependent *S.* Typhimurium macrophage cytotoxicity and *P. syringae*’s hypersensitivity response (HR) in tobacco, furthering its applicability as a broad spectrum T3SS inhibitor [178]. There has also been work detailing the structure-activity relationship (SAR) of thiazolidinones, indicating that although HBF1 has broad activity, it can be designed to specifically act on pathogens of interest [178,179].

### 3.3. Cytoplasmic Complex: Prevention of ATPase Function

**Figure 14:**
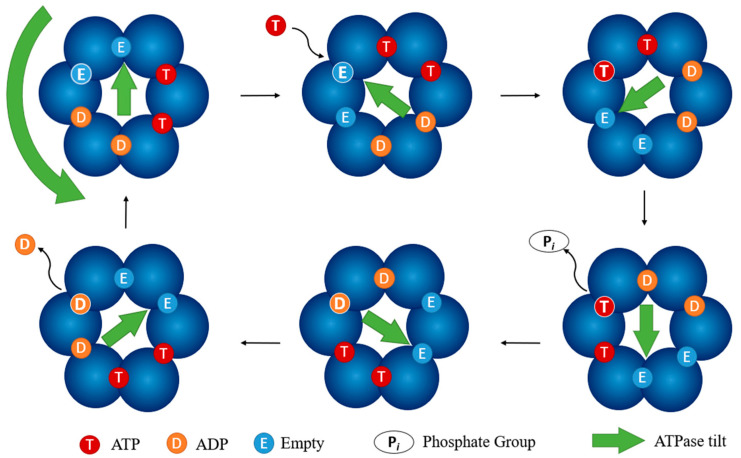
ATPase hexamer cycle of dephosphorylation. The tilt of the hexamer causes unfolded proteins to be “screwed” upwards through the channel and into the needle. Modified from [186].

The ATPase of the cytoplasmic complex powers secretion of effector proteins through the T3SS [38,169,180]. It is a hexamer arranged into a pore that has a diameter of approximately 20 Å. Of the six ATP binding sites on the hexamer, two are filled with ATP, two with ADP, and two are empty. The ATPase powers secretion by tilting upwards at the empty binding sites and rotating this tilt as the ATP is dephosphorylated creating a “screwing” motion (Figure 14). This causes the unfolded effectors to then be pushed upwards through the channel and into the export apparatus, then the needle [38]. The ATPase of the *Salmonella* T3SS-1, InvC, has been implicated as an “unfold-ase” that serves to linearize effectors in an ATP and substrate-dependent manner [125,181]. The conserved structure of the T3SS family of ATPases suggests this may be a universal role for SctN [182]. Without the ATPase, the T3SS is nonfunctional as seen in ATPase knockouts [183,184,185]. It is so necessary for virulence that one *Y. pestis* ATPase knockout strain has been tested as a potential live, attenuated vaccine candidate. The *YscN* mutant strain was shown to prevent the secretion of LcrV, the needle tip, as well as showing dose-dependent protection against *Y. pestis* challenge in mice [185].

**Figure 15:**
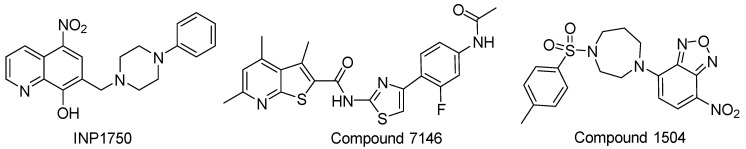
Structures of INP1750, compound 7146, and compound 1504.

The development of T3SS inhibitors that target the ATPase is one of the best-studied targets. Inhibitors have been found against multiple species of bacteria, including *Burkholderia* spp. [187], *Chlamydia* spp. [188,189], EPEC [190], *Pseudomonas* spp. [191,192,193], *Shigella* spp. [194,195], and *Yersinia* spp. [184,196]. Of these inhibitors, one class of small molecule, hydroxyquinolines, has been shown to inhibit the ATPase of T3SS in multiple species. Compound INP1750, an 8-hydroxyquinoline derivative (Figure 15), was originally thought to inhibit the needle tip of *Y. pseudotuberculosis* and *C. trachomatis* but has since been shown to act on the ATPase in *P. aeruginosa*, *Y. pseudotuberculosis,* and *C. trachomatis* [50,116,191,193].

Swietnicki et al. performed a virtual screen of approximately 2,000,000 compounds to determine their binding potential to the *Y. pestis* ATPase, YscN. From this screen, they purchased 50 compounds to test in vitro. Compounds 7146 and 1504 (Figure 15) were able to inhibit YscN’s ATPase activity at IC_50_ with 16.5 and 18.2 µM concentrations, respectively [184]. They also tested the ability of these compounds to inhibit *Burkholderia mallei*’s ATPase, BscN, as it shares 40% identity with YscN [184,185,187]. Compound 7146 had a lower IC_50_, 7.4 µM, while compound 1504′s IC_50_ was increased at 28.4 µM [184]. This demonstrates the high specificity of ATPase binding sites and the possibility to design inhibitors of specific ATPases. Compound 939 from the virtual screen was later tested by Gong et al. in *B. pseudomallei* and it inhibited the secretion of *Burkholderia* effector, BopE, and reduced intracellular survival in RAW 264.7 cells (murine macrophages) [187].

**Figure 16:**
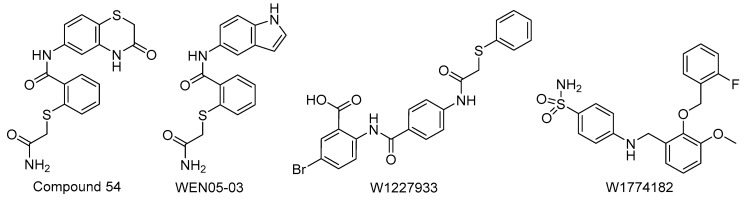
Structures of Compound **54**, WEN03-05, W1227933, and W1774182.

The WEN compounds, a new class of small-molecule inhibitors derived from compound **54** (Figure 16), were discovered using a virtual screen for potential compounds to target the EPEC ATPase, EscN [190]. The screening campaign was conducted using the ATP binding site of EscN (PDB code: 2OMB) and compounds from the ZINC database. The initial 3000 compounds were filtered for predicted cytotoxicity, and the remaining top 70 compounds were purchased for ATP hydrolysis screening using recombinant EscN. Next, compounds were screened for their cytotoxicity toward HeLa cells. Finally, five compounds were screened for their ability to inhibit the T3SS based on the relative secretion of effector EspG1 according to Western blot results. Analogs of one compound were designed based on the predicted binding pose within the EscN active site. The most efficacious compound was WEN05-03, with a K_i_ = 16 ± 2 μM (Figure 16) [190].

Grishin et al. performed a virtual screen to discover compounds that bind to the ATPase of *C. trachomatis*, CdsN. From the 221,574 compounds screened, 16 were purchased for in vitro testing, having eliminated compounds for poor binding scores as well as non-favorable Lipinski characteristics, a set of common rules used in the small molecule drug discovery process (including guidelines on parameters such as the number of hydrogen bond donors and molecular weight) [197]. The most potent compounds, W1227933 and W1774182 (Figure 16) decreased the number of chlamydial inclusions in McCoy cells by 70% and 90%, respectively, when incubated at 50 µM. The inclusions that were present were also much smaller in size compared to control inclusions. When McCoy cells infected with *C. trachomatis* were incubated with either inhibitor at 25 µM for 24 h, they reduced the secretion of effector IncA to undetectable levels [188]. Disruption of the interaction between CdsN and the linker protein, CdsL, by peptide mimetics has also been shown to act as an effective secretion inhibitor. W1227933 and W1774182 also inhibited *C. pneumoniae* invasion of HeLa cells dose-dependently [189]. This indicates inhibition of ATPase binding can be similarly potent as ATPase activity inhibition.

## 4. Inhibition of Effectors

T3SS inhibitors have a vast array of possible activities and targets, the most common of which are placed into four larger categories: interference with the host cytoskeleton, cellular trafficking, cell death and/or survival pathways, and NF-κB/MAPK pathways [67]. Each of these categories can be either up or downregulated to varying degrees based upon the pathogen in question. The prospect of inhibiting a single inhibitor can also seem reductive as many effectors have redundancy in their activity and a singular knockout will not cause complete attenuation of virulence. Notwithstanding this, there are exceptions in which a particular effector is responsible for most of the virulence or host-wide symptoms [198]. In these cases, targeting effector proteins is a possible course of action.

**Figure 17:**
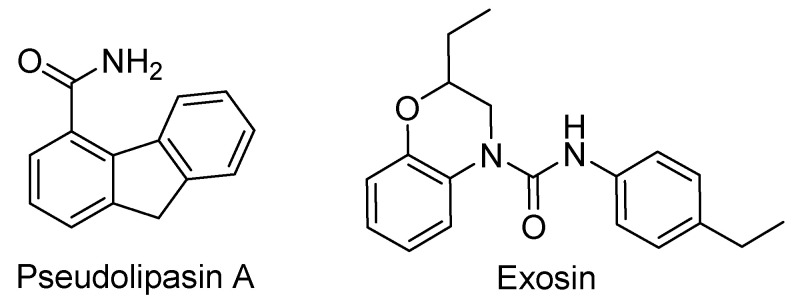
Structure of pseudolipasin A and exosin.

The T3SS effector ExoU is a cytotoxin associated with up to 90% of severe cases of *P. aeruginosa* infections [198]. ExoU’s phospholipase A2 activity is tied to pathogenicity via its interaction with host cell superoxide dismutase 1 inducing cell lysis of the target cell, as well as attenuating particular cellular signaling pathways [199,200]. 9H-fluorene-4-carboxamide, designated pseudolipasin A (Figure 17), is a specific inhibitor for the phospholipase A2 activity of *P. aeruginosa* ExoU. It was shown to not have any effect on T3SS secretion or formation, but rather inhibited the activity of the cytotoxin [201]. Arylsulfonamides were identified as ExoU inhibitors during a screen to identify compounds inhibiting ExoU-dependent cytotoxicity in yeast [202]. A small SAR was performed and although none of the arylsulfonamide derivatives were as potent as pseudolipasin A, it indicated this scaffold could be used to develop inhibitors [200].

ExoS is another important T3SS effector/toxin in *P. aeruginosa*. It contains a membrane localization domain to assist in delivery to the membrane in a cholesterol-dependent manner. The ADP-ribosyltransferase activity of ExoS can inhibit Rab 5 and Ras2p function [203,204]. ExoS also contains a GAP domain whose action disrupts the actin cytoskeleton, inhibits bacterial internalization into macrophages, induces host cell rounding, and prevents wound healing [199]. Key mutations in ExoS’s binding domain significantly affect its ability to cause death in a mouse model of pneumonia, making it an attractive inhibitory target [198]. Exosin (Figure 17) has been identified as a competitive inhibitor of ExoS ADP-ribosyltransferase activity. It, as well as multiple derivatives, confers protection of CHO cells from ExoS-induced cytotoxicity with the most potent analog giving nearly 40% recovery [204].

### 4.1. Adhesion Inhibition

The T3SS is a contributor to the ability of bacterial cells to adhere to eukaryotic host cells, primarily in the translocon-needle tip interaction. There are, however, numerous other mechanisms, both T3SS-dependent and independent, that contribute to the majority of the adhesion process. Using pathogenic *E. coli* as an example, we will discuss the T3SS-dependent adhesion mechanism, called intimate attachment, to then show examples of intimate adhesion inhibition as it is the best characterized. As mentioned, there are other methods of attachment related to the T3SS and its effector proteins in different species of bacteria. These methods/mechanisms can be inhibited with similar overall outcomes [87,89,205,206,207,208,209,210,211].

Some analyses into the ability of compounds to inhibit cellular adhesion have been performed to complement screens of potential T3SS inhibitors. These assays typically involve fluorescence microscopy to visualize the adherence of the bacteria to the cell surface. Since EPEC, EHEC, and *C. rodentium* (a well-adopted mouse model for EPEC/EHEC infection) [212] are attaching and effacing pathogens, pedestal formation and changes in eukaryotic morphology may also be noted. It is noteworthy that although these compounds inhibit adhesion, and therefore the systemic infection, they don’t inhibit T3SS secretion. The effectors are still secreted into the extracellular matrix and can cause minor effects to the host cells.

**Figure 18:**
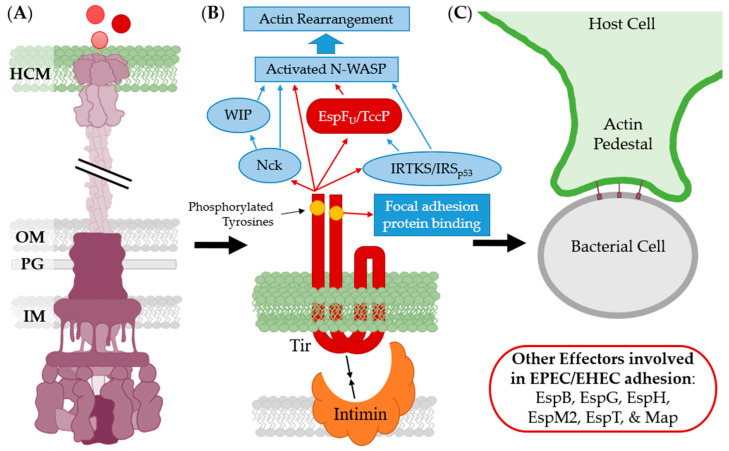
Process of intimate adhesion via Tir-intimin binding in pathogenic *E. coli*. (**A**) Pathogen contacts the host cell via T3SS and secretes effectors (red), including Tir; (**B**) Tir incorporates into the host membrane and binds to intimin on the bacterial membrane. The N-terminus of Tir binds to focal adhesion proteins within the host. Tir’s C-terminus interacts with multiple host factors (blue) to cause actin rearrangement; (**C**) Actin rearrangement causes the formation of the characteristic actin pedestal around the bacterial cell. Modified from [213]. Abbreviations: OM, outer membrane; PG, peptidoglycan; IM, inner membrane; HCM, host cell membrane.

The translocated intimin receptor (Tir) is one of the first effector proteins translocated by *E. coli*’s T3SS into host cells and is involved in the intimate binding process [213,214,215]. There are multiple other effector proteins, including EspB, EspG, EspH, EspM2, EspT, Map, and TccP, that assist in adhesion via cytoskeleton rearrangement in redundant or similar mechanisms to Tir (Figure 18). Knockouts of these effectors are not as attenuated as Tir knockouts indicating the interaction between intimin and Tir is the most important for intimate attachment. Mutational studies confirm that changes to the receptor recognition domain abolish attachment capabilities [216]. Tir integrates into the host membrane after translocation and its N- and C-terminal tyrosines are phosphorylated by host kinases [27]. As intimin binds to the extracellular loops of Tir, the phosphorylated tyrosines act upon focal adhesion proteins, including α-actinin [217], host pathways (Ex. Nck and IRTKS/IRSp53), and effectors (Ex. EspF) [218,219,220,221]. This results in pedestal formation and intimate attachment [213,215,217].

Due to Tir’s extracellular localization, inhibitors can bind without having to cross the eukaryotic membrane. Girard et al. found that anti-Tir IgY was effective at preventing bacterial adhesion to porcine ileal in vitro organ culture against both porcine and human strains of enteropathogenic *E. coli* (EPEC). Ruano-Gallego et al. assessed the potential of an anti-Tir nanobody, TD4, as a treatment or prophylactic for EHEC infections. TD4 inhibits the attachment of EHEC to HeLa cells and reduces adherence to human colonic mucosa [222]. The Tir-binding domain of intimin has also been investigated as an indirect way to inhibit Tir adhesion. Anti-intimin IgY was effective at reducing the adherence of both EPEC strains to host gut epithelial tissue in an ileal loop assay as well as in oral administration of the IgY [223]. Saberianfar et al. investigated the Tir-binding domain of intimin as a target to isolate sdAbs from tobacco leaves. These sdAbs were used to design a chimeric Ab, V_H_H10-IgA. This Ab inhibited four strains of EHEC from adhering to host cells, with three of the four completely inhibited [224]. V_H_H10-IgA’s cross-serotype inhibition of bacterial adhesion is highly promising for future studies.

**Figure 19:**
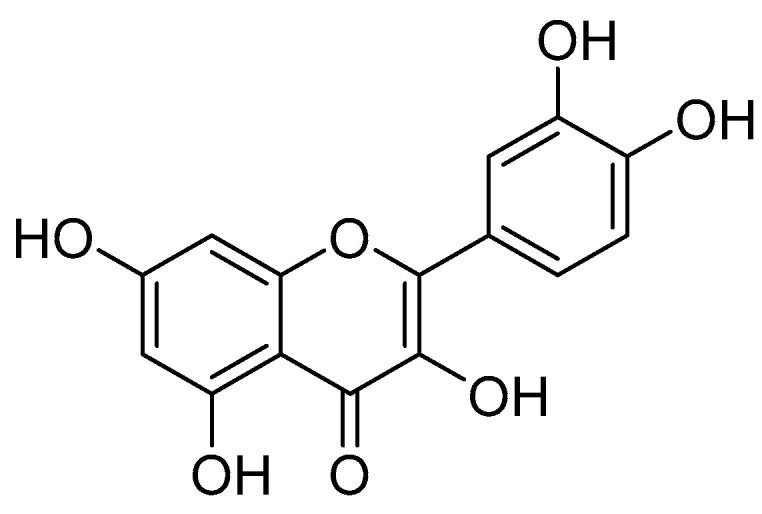
Structure of Quercetin.

Multiple other small molecule and antibody inhibitors of adhesion targeting the intimate attachment of pathogenic *E. coli* have been discovered through the years [102,122,225,226,227,228,229,230,231]. Quercetin (Figure 19) was recently implicated as an inhibitor by Xue et al. while observing *E. coli* O157:H7, the EHEC strain most commonly causing outbreaks, adhesion to human colon adenocarcinoma-2 (Caco-2) cells. Quercetin attenuated the association of multiple focal adhesion proteins, implying a mechanism of action associated with the N-terminus of Tir [232]. Lin et al. tested quercetin’s ability to reduce infection in a *C. rodentium* mouse model. They found that quercetin reduced colitis severity in infected mice, but the cause could not be confidently attributed to its adhesion inhibitory properties. This is because the populations of commensal bacteria such as *Bacteroides*, *Bifidobacterium*, *Lactobacillus*, and *Clostridia*, were increased in quercetin-treated mice. These commensals were likely able to out-compete the *C. rodentium* in the colon [233].

### 4.2. Internalization Inhibition

Internalization is not the mechanism employed by all T3SS encoding bacterium, but it is essential for those that do. As with adhesion, one pathogen will be used to explain and exemplify the possible outcomes of internalization inhibition. *S*. Typhimurium is notorious for its ability to invade multiple mammalian and plant cell types [234,235,236]. *S*. Typhimurium has multiple redundant mechanisms for adhesion and invasion, some T3SS-dependent, and some T3SS-independent. The only internalization via the T3SS allows for intracellular proliferation [237,238].

**Figure 20:**
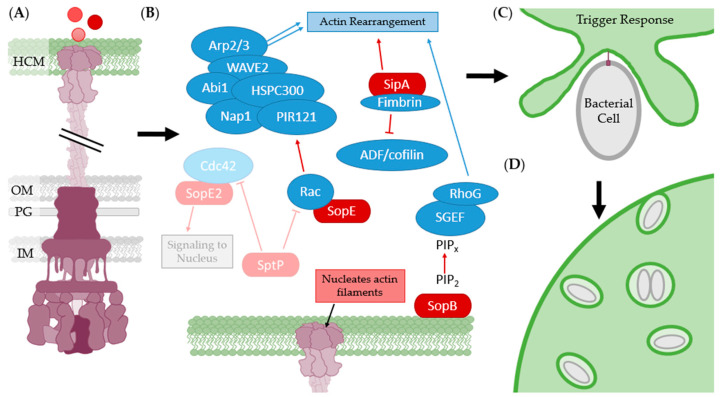
Process of T3SS-1-related trigger internalization in *S. enterica*. (**A**) Pathogen contacts the host cell via T3SS-1 and secretes effectors, including SipA, SopE, SopE2, SopB, and SptP; (**B**) These effectors (red) interact with numerous host factors (blue) that lead to actin rearrangement and polymerization. SipC acts as a nucleus for actin filaments, with growth outwards from the pore; (**C**) Actin rearrangement causes the formation of membrane ruffling that then engulfs the bacterial cell; (**D**) Once within the SCV, it expresses T3SS-2 and begins proliferation. Multiple bacterial cells can be internalized within a single host cell. Modified from [238]. Abbreviations: OM, outer membrane; PG, peptidoglycan; IM, inner membrane; HCM, host cell membrane.

First, the T3SS-1 makes contact with the host cell and injects multiple effector proteins including SipA, SopE, SopE2, SopB, and SptP (Figure 20A). SipA binds to fimbrin, promoting actin rearrangement and polymerization as well as stabilizing the new actin filaments [239,240,241,242]. The minor translocon component, SipC, nucleates the actin filaments that grow out from the pore and create the membrane ruffles [238,243]. SopE activates Rac which eventually leads to upregulation of Arp2/3 and branching of the new actin filaments [240,244]. SopB upregulates the conversion of PIP_2_ to PIP_x_, thereby increasing activation of RhoG and membrane ruffling, called the trigger response [245,246]. Once the host membrane ruffles have engulfed the bacterial cell into a *Salmonella* containing vesicle (SCV), SptP then downregulates Rac and Cdc42 via GAP activity to reduce host membrane ruffling (Figure 20B, faded) [247,248]. *S. Typhimurium* then expresses its T3SS-2 to proliferate and maintain its intracellular lifestyle (Figure 20D).

**Figure 21:**
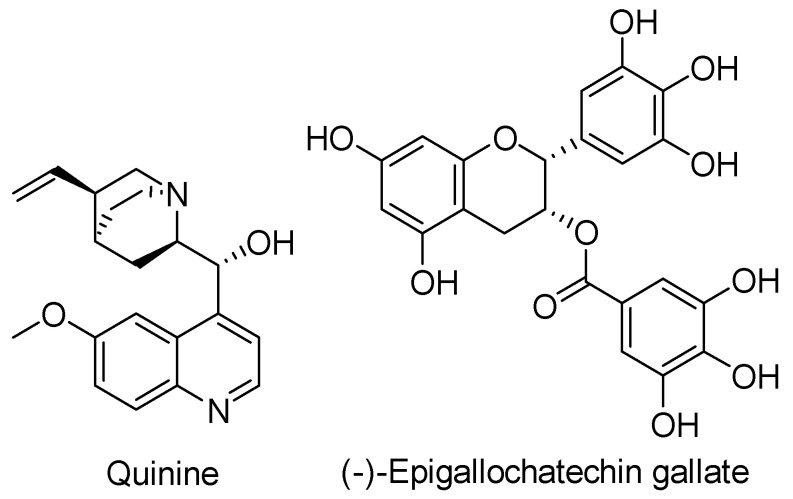
Structure of quinine and (-)-epigallocatechin gallate (EGCG).

Quinine, an FDA-approved anti-malaria drug (Figure 21), was shown to inhibit the invasion process of *S. Typhimurium* and *Shigella flexneri* [249]. Wolf et al. analyzed the ability of these pathogens to invade Caco-2 cells in the presence of 50 and 100 µM quinine sulfate. Extracellular bacteria were destroyed via gentamicin addition and intracellular bacterial were enumerated after lysis of the infected Caco-2 cells. Both pathogens were inhibited in a dose-dependent manner, although the highest concentration was only one order of magnitude smaller than the control. Cytotoxicity and growth inhibition tests showed that inhibition was occurring and not cell death. These results have been called into question, however, by the work of Kharal et al. who showed quinine was bactericidal to multiple species of bacteria, including *P. aeruginosa* and *S. enterica* serovar Typhi [250].

The mechanism of host cell invasion and adhesion by pathogenic bacteria are often related. Inhibitors of the internalization process will potentially be adhesion inhibitors and vice versa. EGCG (Figure 21) has been shown to inhibit T3SS-mediated adhesion and internalization of multiple organisms including EPEC and EHEC, *S.* Typhimurium, and *Y. pestis* [251,252]. EGCG was analyzed for its ability to inhibit adherence of EPEC to human epithelial type 2 (Hep-2) cells. In this assay, EPEC was cultured with the Hep-2 cells and incubated for 2 h in the presence and absence of EGCG. The Hep-2 cells were then affixed to a microscope slide and stained with Giemsa to determine the number of adhered bacteria. EGCG at concentrations of 100 μM reduced adherence by ~50% [251]. Tsou et al. evaluated EGCG’s ability to inhibit *S*. Typhimurium invasion into HeLa cells via flow cytometry and immunofluorescence imaging using anti-*Salmonella* antibody staining. *S*. Typhimurium grown in the presence of 100 µM EGCG showed a significant reduction in its ability to invade the cultured HeLa cells. The level of reduction was similar to that of *S*. Typhimurium strain with a nonfunctional InvA. They demonstrated that EGCG did not affect bacterial growth but was inhibiting SPI1 T3SS-dependent *S*. Typhimurium invasion of host cells [252].

### 4.3. Effector Chaperone Inhibition

Effector chaperones come in two varieties, class IA and class IB. Both IA and IB chaperones typically bind to the N-terminus of the effector by winding the unfolded effector around itself. There has also been some research showing the C-terminus of the effector retaining a globular conformation. This is commonly referred to as “threading the needle” [128]. Class IA chaperones also typically only have one binding partner, while class IB chaperones have multiple unique effectors. Another differentiating factor between class IA and IB is that genes for IA chaperones are encoded adjacent to their binding partner, while IB chaperones are encoded near structural components.

**Figure 22:**
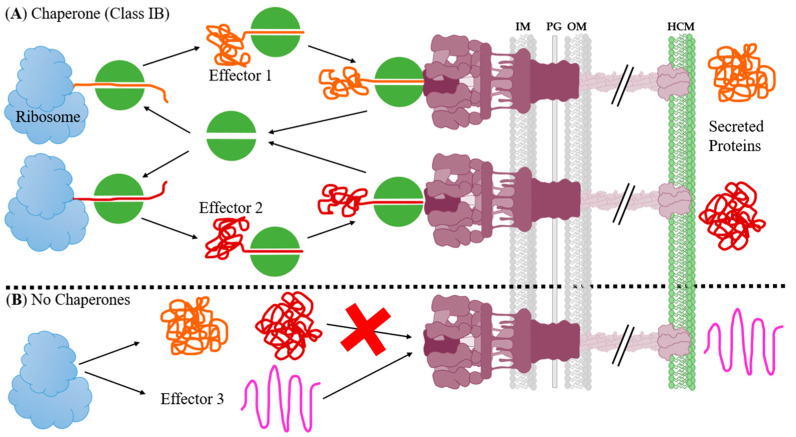
T3SS chaperone protein function. (**A**) Class IB chaperone protein (green) assisting effectors (orange and red) in secretion. (**B**) Without the chaperone, the substrate effectors cannot be secreted. There are some effectors (Effector 3, pink) that do not require chaperones and will be secreted. Modified from [58]. Abbreviations: OM, outer membrane; PG, peptidoglycan; IM, inner membrane; HCM, host cell membrane.

The chaperone holds the effector and prevents the CBD from folding as it is transcribed by the ribosome. The chaperone carries the effector until it deposits it into the sorting platform of the basal body, allowing for a successful secretion of the linear effector. The chaperone then returns to the ribosome to cycle through the process again. In the case of a class IB chaperone, the cycle can continue with the same or an altogether different effector (Figure 22A). Knockouts of these multi-substrate chaperones, such as CesT, can drastically reduce or even eliminate the virulence capabilities of the pathogen. In the case of CesT, Runte et al. showed mutations in either tyrosine 152 or tyrosine 153 to phenylalanine had delayed colonization and the strains were more easily cleared from the intestine in a *C. rodentium* model of infection [253].

Similar to structural chaperone inhibition, the inhibition of effector chaperones will have similar effects to inhibiting the effector itself. This comes with the caveat that class IB chaperones can have multiple effectors as binding partners [254]. In essence, this leads to the inhibition of multiple effectors with one inhibitor. For example, the EPEC chaperone CesT has at least 13 known substrates: Tir, Map, EscJ, EscC, EspF, NleA, NleG, NleH1, NleH2, NleF, EspH, EspZ, & EspG, making it an attractive target for inhibition [127,253,255,256,257,258,259,260]. As with the effector inhibition, there is the complication that even if you inhibit all effector chaperones within a pathogen then there will still be some effectors that are self-chaperoning and/or membrane-permeable (Figure 22B) [133,261]. They are often “floppy” or less structured proteins. An example of this would be the membrane-permeable effector from *Y. pestis*, YopM [262,263].

Targeting the protein-protein interaction between SctU and the CBD of chaperones is an attractive notion. In the absence of a correctly folded SctU, there was an increase of chaperone accumulation on the bacterial inner membrane, suggesting the interaction between the chaperone and SctU is necessary for T3SS secretion, making it an attractive interaction to target [167]. Also, targeting the CBD will potentially inhibit self-chaperoning proteins as well as chaperones as they share a common motif [133]. To date, there have been no inhibitors shown to interact at this site, likely due to the lack of mechanistic studies on effector secretion inhibitors.

## 5. Conclusions

The number of identified inhibitors of the T3SS has steadily increased over the years, but they are often discovered in phenotypic assays. This results in a lack of knowledge regarding their mechanism of action and the binding partners of the inhibitors. The current minimalistic approach to determining the mechanism of action of T3SS inhibitors can lead to incorrect assumptions about the target. The SAs are the perfect example of this effect. They have been shown to inhibit many structural features of the T3SS, but it is not yet confirmed if this is due to the small structural changes in the compounds screened, or due to bias of the phenotypic screen. Many SAs have also been shown to modulate the expression or genetic regulation of T3SS components and effectors – a possible explanation for the disparity in phenotypic results. Although these inhibitors, like INP0341 [264], have shown recent successes in vitro and in vivo, they were excluded from this review as they have yet to be shown as direct inhibitors of the T3SS.

This review covered direct inhibitors of T3SS components, whether structural or functional in mechanism. Inhibition of needle and translocon components, and/or their regulatory proteins, reduces the number of functional needles and increases truncated needle prevalence, resulting in little to no effector translocation into host cells, but can allow for secretion into the intracellular matrix. Inhibitors binding to any components of the T3SS base, including the basal body, export apparatus, and cytoplasmic complex, tend to prevent the formation of functional T3SS apparatuses, thereby stopping effector secretion. While this a more complete method of inhibition, inhibitors using this method are susceptible to off-target effects as base components of the T3SS are more conserved with other secretion systems. Effector inhibition, while not highly effective for most T3SS-utilizing pathogens, is very effective when a specific effector is responsible for a majority of symptoms or pathogenesis initiation. Collectively, this knowledge can be used for the rational design and improvement of T3SS inhibitors.

## Figures and Tables

**Table 1:** Sequences of effectors and synthesized coiled-coil peptides. Red residues were changed to increase solubility. Greyed sequences are original to the protein.

Name	Sequence
EspA	KANNLTTTVNNSQLEIQQMSNTLNLLTSARSDMQSLQYRTISGI
Coil A	LTTTVNNSQLEIQQM
Coil B	MSNTLNLLTSARSDM
Coil AB1	KMNNLTTKVNNLQLELQEMRNTLKRLKSAMRRMQ
Coil AB2	KINNLTTKVNNLQLELQEMRNTLKRLKSAMRRMQ
EscF	LSDSVPELLNSTDLVNDPEKMLELQFAVQ
Coil C	LSDSVPELLNSTDLV
Coil D	VNDPEKMLELQFAVQ
CesA	IVSQTRNKELLDKKIRSEIEAIKKIIAEFDVVKESVNELSEKAK
Ces A1	IVSQTRNKELLDKKIRSEIEA
Ces A2	IKKIIAEFDVVKESVNELSEK

**Table 2:** Select class II and III chaperones.

Species (T3SS)	Chaperone	Binding Partner	Class	PDB/REF
*Aeromonas* spp.	AcrH	AopB & AopD	II	3WXX [134]
	AscE/AscG	AscF	III	3PH0 [135]
EPEC/EHEC	CesD	EspB & EspD	II	[136,137]
	CesD2	EspD	II	[138]
	EscE/EscG	EscF	III	[129]
	CesA (CesAB)	EspA	III	1XOU (2M1N) [130,131]
	EscG/CesA2	EscF & EspA	III	[129,130,139]
*Pseudomonas* spp.	PcrH	PopB & PopD	II	2XCC, 2XCB [140]
	PcrG	PcrV	III	[141]
	PscE/PscG	PscF	III	2UWJ [142]
*Salmonella* spp. (SPI1)	SicA	SipB & SipC	II	[143]
	SipD	PrgI	III	3ZQB [144]
*Salmonella* spp. (SPI2)	SseA	SseC & SseD	II	[145]
	SseA	SseB	III	[146]
	SsaH/SsaE	SsaG	III	[147]
*Shigella* spp.	IpgC	IpaB & IpaC	II	3GYZ [148]
	Spa13	MxiH	III	[149]
*Yersinia* spp.	SycD	YopD & YscO	II	4AM9 [150]
	LcrG	LcrV	III	[151]
	YscE/YscG	YscF	III	2P58 [152]

## Data Availability

Not applicable.
